# Effect of ubiquinol supplementation on biochemical and oxidative stress indexes after intense exercise in young athletes

**DOI:** 10.1080/13510002.2018.1472924

**Published:** 2018-05-08

**Authors:** Patrick Orlando, Sonia Silvestri, Roberta Galeazzi, Roberto Antonicelli, Fabio Marcheggiani, Ilenia Cirilli, Tiziana Bacchetti, Luca Tiano

**Affiliations:** a Department of Life and Environmental Sciences, Polytechnic University of Marche, Ancona, Italy; b Clinical and Molecular Diagnostic Laboratory, INRCA-IRCCS National Institute, Ancona, Italy; c Department of Cardiology, INRCA-IRCCS National Institute, Ancona, Italy; d Department of Clinical and Dental Sciences, Polytechnic University of Marche, Ancona, Italy

**Keywords:** Intense exercise, oxidative stress, ubiquinol, physical performance, mitochondrial function, reactive oxygen species, paraoxonase activity, peripheral blood mononuclear cells

## Abstract

**Objectives:** Physical exercise significantly impacts the biochemistry of the organism. Ubiquinone is a key component of the mitochondrial respiratory chain and ubiquinol, its reduced and active form, is an emerging molecule in sport nutrition. The aim of this study was to evaluate the effect of ubiquinol supplementation on biochemical and oxidative stress indexes after an intense bout of exercise.

**Methods:** 21 male young athletes (26 + 5 years of age) were randomized in two groups according to a double blind cross-over study, either supplemented with ubiquinol (200 mg/day) or placebo for 1 month. Blood was withdrawn before and after a single bout of intense exercise (40 min run at 85% maxHR). Physical performance, hematochemical parameters, ubiquinone/ubiquinol plasma content, intracellular reactive oxygen species (ROS) level, mitochondrial membrane depolarization, paraoxonase activity and oxidative DNA damage were analyzed.

**Results:** A single bout of intense exercise produced a significant increase in most hematochemical indexes, in particular CK and Mb while, on the contrary, normalized coenzyme Q_10_ plasma content decreased significantly in all subjects. Ubiquinol supplementation prevented exercise-induced CoQ deprivation and decrease in paraoxonase activity. Moreover at a cellular level, in peripheral blood mononuclear cells, ubiquinol supplementation was associated with a significant decrease in cytosolic ROS while mitochondrial membrane potential and oxidative DNA damage remained unchanged.

**Discussion:** Data highlights a very rapid dynamic of CoQ depletion following intense exercise underlying an increased demand by the organism. Ubiquinol supplementation minimized exercise-induced depletion and enhanced plasma and cellular antioxidant levels but it was not able to improve physical performance indexes or markers of muscular damage.

## Introduction

Clinical studies suggest that the beneficial effects of regular physical exercise rely on the intensity of the work-load performed [1–3]. While mild workload may not be sufficient to produce significant ergogenic adaptive effects, workload exceeding the subject-specific threshold could be associated with inefficient recovery leading to a decline in physical performance, a condition also known as overtraining. In both conditions, reactive oxygen species (ROS) represent key modulators of cellular physiology triggering adaptive responses when produced in limited amounts but detrimental when they are produced in excess, leading to oxidative stress and cellular dysfunction [4]. During aerobic exercise the request for oxygen from the skeletal muscle increases 10–20 fold [5]. Enhanced metabolic rate together with a rise in temperature and decrease in cellular pH could accelerate ROS production [6], particularly in mitochondria, where constitutively 1–2% oxygen is converted into superoxide anion [7]. As a result, the exercising muscle produces much larger amounts of ROS compared to the muscle at rest [8,9]. This overproduction in trained individuals is easily neutralized in a dynamic equilibrium. On the contrary, in conditions of intense exercise and overtraining, ROS production can lead to the oxidation of important biological macromolecules such as DNA, polyunsaturated fatty acids, aminoacids and proteins [10,11]. Moreover, the activities of different oxido-reductases (such as xanthine oxidase and NADPH oxidase) seem to play an important role in promoting oxidative stress in muscle cells during exercise [12]. On the other hand regular physical exercise stimulates endogenous antioxidant defense enzymes such as superoxide dismutase and glutathione peroxidase [13] representing *de-facto* an ideal antioxidant [14,15].

In this context it is important to evaluate the optimal workload in order to maximize beneficial effects of physical exercise thus preventing the deleterious ones. To correctly address this issue, it is pivotal to consider the individual characteristics of exercise practitioners. Particular attention should be devoted to professional athletes subjected to elevated workloads but also to regular exercise practitioners characterized by an altered oxidative balance (elderly, dismetabolic patients, etc.). In these cases endogenous antioxidant defenses might not be sufficient to guarantee an optimal response and dietary supplementation with antioxidant and ergogenic substances might be required [16,17].

The topic of antioxidant supplementation in sport nutrition is highly debated. Recent studies underline how antioxidant supplementation might result in down-regulation of ROS-associated hormetic signal [18–20]. However it is important to note that antioxidants represent an extremely heterogenous class of molecules with different chemical–physical properties, cellular localization and biological activities. It is also worth underlining that, while the antagonistic activity of nutritional antioxidants might be sound for healthy subjects undergoing moderate physical exercise, for specific categories of exercise practitioners described above it might be important to investigate when, in what way and what kind of supplementation could be useful to maintain correct oxidative equilibrium.

In the present study we investigated the effects of oral supplementation with ubiquinol, the reduced and active form of Coenzyme Q_10_, in athletes subjected to intense exercise. Coenzyme Q_10_ is an endogenous lipophilic quinone that constitutes a key component of the mitochondrial respiratory chain but it is also present in other cellular sub-fractions and in plasma lipoproteins, where it exerts an important antioxidant role in synergism with vitamin E [21]. Moreover, it is also known to affect gene expression [22]. CoQ_10_ role in sport nutrition has been investigated in several studies showing contrasting results. Malm et al. [23] have shown that following aerobic exercise on a cycloergometer, the placebo group had an improvement in performance over the group supplemented with 120 mg/day of CoQ_10_ for 22 days. Other studies [24,25]observed no significant effects on performance, while Cooke et al. [26], Gokbel et al. [27] and Kon et al. [9] reported positive effects of supplementation resulting in improved performance, prolonged exhaustion time and decrease in fatigue in sedentary and trained subjects undergoing different workloads.

All the studies mentioned above used the oxidized form of CoQ_10_ (ubiquinone). Although the organism is able to efficiently reduce dietary CoQ_10_ the use of the reduced form as a supplement has several advantages: it has greater bioavailability and is readily usable by the organism since reductive steps are not required [28]. This is of particular relevance in conditions when reductive systems might be less efficient such as during ageing or following intense exercise. The development of a stable, reduced form of CoQ_10_ available as oral supplement is quite recent and, therefore, the number of scientific reports on its use are still limited, particularly regarding sport and nutrition.

The aim of the present study was to evaluate the effect of ubiquinol supplementation on biochemical and oxidative stress indexes after an intense bout of exercise in trained athletes. In particular we focused on the Coenzyme Q10 plasma content and its oxidative status, activity of the antioxidant and anti-inflammatory enzyme paraoxonase-1, and muscle damage indexes (plasma CK and Mb); moreover we evaluated the effect of 200 mg/day ubiquinol supplementation for 1 month on the biochemical profiles modulated by physical exercise. As a secondary endpoint we also quantified the effects of supplementation on indexes of physical performance.

## Materials and methods

### Study design

**Figure 1.**
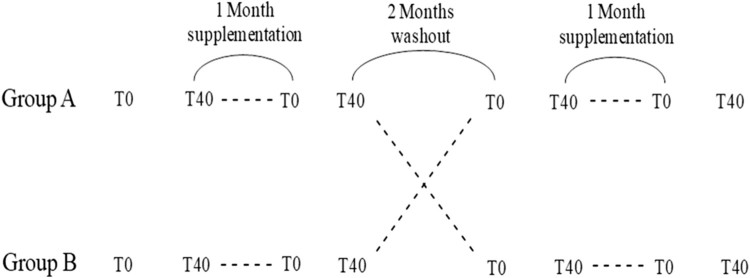
Study design. T0 = base line; T40 = after a single bout of intense exercise at 85% of the maximal heart rate (HRmax).

Subjects eligible for recruitment to the study were healthy trained males. In particular, 21 male athletes from Stamura Rugby Team Ancona (age 26 ± 5; BMI 25 ± 4), were enrolled and randomized for a double blind cross-over controlled study (Figure 1). The study lasted 8 months and during this period the level of training was constant (three training sessions and one game per week). None of the participants were on any prescribed medication or taking any dietary supplements within 1 month before the start of the study. Subjects were randomized at the beginning of the study to either 200 mg ubiquinol/day or to placebo (soya lecithin, medium chain triglyceride) for 1 month, once a day with one of the main meals. After the first month of supplementation, both groups underwent a 60 day washout period followed by a crossover phase, where ubiquinol/placebo groups were switched. Dosage and time of supplementation are in line with previous reports on the use of CoQ_10_ in sport nutrition [9,26] and 200 mg is the accepted maximal daily dose as a food supplement in Italy. The study was approved by INRCA Ethical Committee and conducted in agreement with the Helsinki Declaration on human research. Informed consent was obtained from all study participants.

### Physical performance

Each session of intense exercise was conducted at least 48 hrs after the last training or game, and the first experimental session was conducted after two months of standardized training of the team. A single bout of intense exercise, defined as 40 min run on a treadmill, was set at a speed able to produce a heart rate (HR) in volunteers equivalent to 85% of the maximal heart rate (HRmax). HR was monitored using Polar H10 heart rate sensor device and heart rate reserve (HRreserve), proportional to the percentage of VO_2_ reserve, was calculated according to Karvonen’s formula [29] that takes into account both the subject’s age and basal heart rate (HRrest). Karvonen normalized HRreserve is calculated as the difference between predicted maximum heart rate (220-age) and resting heart rate (HRreserve = HRmax – HRrest). Karvonen corrected 85% HRmax is calculated as [(HRreserve x 0.85) + HRrest]. During the experimental session, the treadmill speed was set at 6 km/h to warm up and subsequently the pace progressively increased by 1 km/h every 60 sec. Within 5–7 min volunteers reached the requested heart rate and from this moment they ran for 40 min. HR and speed were recorded every 5 min and the speed adjusted in order to keep heart rate at 85% of the HRmax. Average speed and time required to reach 75% of the max speed were evaluated.

### Sample collection

During each of the four experimental sessions (at the beginning and end of the supplementation phase) blood was withdrawn from volunteers before and after each single bout of intense exercise.15 mL of peripheral blood was drawn into a heparinized vacutainer. Tubes were centrifuged immediately at 1600 g for 10 min at 4°C to separate plasma which was subsequently kept at −80°C until use for hematochemical analysis, total plasma and oxidative status of coenzyme Q_10_. The hematochemical parameters evaluated were: circulating markers of muscle damage (myoglobin and creatine kinase), creatinine, triglycerides, uric acid, glucose, urea, low-density lipoproteins, high-density lipoproteins, total cholesterol and albumin. The above mentioned analyses were conducted at the hospital laboratory of the National Research Institute on Aging (INRCA), Ancona. In parallel, samples collected in EDTA vacutainer were immediately treated with a cryopreserving solution (human albumin at 5% and 20% and Dimethyl sulfoxide; 3:1:1), quickly frozen in dry ice and stored at −80°C in order to maintain cellular integrity of peripheral blood mononuclear cells (PBMCs). Whole blood cryopreservation methodology was adapted from a previous protocol on isolated cells [30] and was carefully optimized in order to verify stability of the cryopreserved cells over time. Data produced a viability of recovered cells well over 80% and flow cytometric distribution of major endpoints was reproducible over a period of 6 months. Only for post-exercise withdrawal, total blood (15 mL) collected in EDTA, was diluted in RPMI medium (2:1) and incubated at 37°C with gentle shaking to simulate and investigate post exercise recovery of the cells up to 210 min. Within this time, samples were collected at specific time points (90, 150 and 210 min) and cryopreserved as described above. Cellular analyses for all experimental points for each subject were conducted in a single day in order to reduce variability. Finally, 5 mL of serum collected before and after both physical exercise session and supplementation was stored at −80°C until use for paraoxonase 1 activity assay.

### PBMC isolation

At the moment of use, 2 mL of cryopreserved blood was washed once with PBS and immediately used for PBMC isolation by density-gradient centrifugation using lymphoprep (Nyegaard, Oslo, Norway). Isolated cells were washed twice in PBS and counted using a Guava ViaCount kit in flow cytometry (Merck Millipore, Milan, Italy). The assay is able to discriminate viable cells from dead ones and from debris as described by Silvestri et al. [31].

### Coenzyme q_10_ plasma level and its oxidative status

CoQ_10_ content and its oxidative status (ubiquinone) in plasma were assayed by HPLC with electro-chemical detector (ECD) by Shiseido Co. Ltd, Japan. The mobile phase was 50 mM sodium perchlorate in methanol/distilled water (95/5, v/v) with flow rate of 0.2 mL/min. Using a column-switching system, coenzyme was eluted from the concentrating column by mobile phase 2 (50 mM sodium perchlorate in methanol/iso-propanol (90/10 v/v)) with a flow rate of 0.24 mL/min. The column oven was set to 40°C. Pump one and two were model 3001, auto-sampler model 3033, switch valve model 3012, concentration column CQC (C8 DD; 10 mm x 4.0 mm ID) and separation column CQS (C18 AQ; 150 mm x 2.0 mm ID, particle size at 3 µm diameter) were all supplied by Shiseido Co. Ltd. A peculiarity of the system was the use of a post-separation reducing column (Shiseido CQR) capable of fully reducing the peak of ubiquinone. Prediluted CoQ_10_ standard was prepared in ethanol (2.25 µg/mL, max concentration) and stored at −80°C until use. The oxidation potential for ECD was 650 mV. Plasma levels of CoQ_10_ were normalized by total cholesterol content (nmol CoQ_10_/mmChol), while levels of oxidation were expressed as percentage of ubiquinone/total CoQ_10_.

### Intracellular ROS assay

Intracellular ROS levels were assessed in viable PBMC by means of 2′–7′-dichlorodihydrofluoresceindiacetate (DA-DCFH_2_), a non-polar probe, which readily diffuses across cell membranes where it is hydrolyzed by intracellular esterases to the non-fluorescent polar derivative, DCFH_2_. In the presence of ROS, DCFH is oxidized to DCF which is highly fluorescent and whose emission maximum can be monitored at 520 nm. After isolation of PBMC from each sample (t0–40–90–150–210 min), 50,000 cells were incubated with DA-DCFH_2_ (10 µM final concentration) for 30 min at 37°C in the dark. Then, cells were washed in PBS by centrifuging at 600 g for 10 min at 4°C and resuspended in PBS. Fluorescence intensity was recorded only on live cells detected by Guava ViaCount kit (Merck Millipore, Milan, Italy), which contains a mixture of fluorescent dyes able to discriminate viable from apoptotic and dead cells. Five thousand events for each sample were measured. Fluorescence was measured using an excitation wavelength of 488 nm and recording emission at 525/30 (Green) for DCF, 583/26 (Yellow) and 690/50 (Red) for ViaCount. A region representative of high levels of fluorescence was arbitrarily defined using a gate relative to 15% of the population of baseline cells (before physical exercise session and supplementation) in a reference experiment. These settings were then maintained for all subsequent experiments and the relative percentage of cells for the high ROS region was calculated. Results were analyzed using Guava InCyte software.

### Mitochondrial membrane depolarization assay

Mitochondrial membrane depolarization was evaluated in viable PBMC by use of the MitoSense Red dye kit (Merck Millipore, Milan, Italy), a fluorescent cationic dye that accumulates in the mitochondria and its fluorescence is proportional to mitochondrial membrane potential according to Nernst’s law. After isolation of PBMC from each sample (t0–40–90–150–210 min), 50.000 cells were incubated with MitoSense Red (Flow Cellect^TM^ MitoDamage Kit) for 15 min at 37°C in the dark. Then, cells were washed twice in assay buffer 1X by centrifuging at 600 g for 5 min at room temperature and resuspended in assay buffer 1X. MitoSense Red was excited with a red laser (638 nm) and emission was recorded at 658 nm (Red2 fluorescence). Results were analyzed using Guava InCyte software.

### Paraoxonase 1 (PON1) activities assay

PON1 activity was evaluated using two different substrates, paraoxon for paraoxonase activity and phenylacetate for arylesterase activity. All assays were performed in a 96 well plate, in a total reaction volume of 200 µL. Paraoxonase activity was evaluated in serum (10 µL, non-diluted samples). The basal assay mixture included 5 mM Tris-HCl, pH 7.4 containing 0.15 M NaCl, 4 mM MgCl_2_, 2 mM CaCl_2_ and 1.0 mmol/L paraoxon. Paraoxon hydrolysis was spectrophotometrically monitored for 8 min (every 15 s) at 412 nm. Non-enzymatic hydrolysis of paraoxon was subtracted from the total rate of hydrolysis. One unit of PON1 paraoxonase activity was equivalent to 1 nmol of paraoxon hydrolyzed/min/mL. Arylesterase activity was analyzed on serum samples that were diluted 1:10 with 1 mmol/L CaCl_2_ in 50 mmol/L Tris HCl, pH 8.0 and then, 5 µL was taken for a total reaction volume of 200 µL. After addition of the substrate phenyl acetate (1.0 mmol/L), the hydrolysis was monitored at 270 nm for 3 min (every 15 s). One unit of arylesterase activity was equivalent to 1 µmol of phenyl acetate hydrolyzed/min/mL. Activities were normalized by HDL content and expressed as U/mg HDL [32].

### Comet assay

Oxidative DNA damage was conducted on cryopreserved cells as described above in according method of Tiano et al. [30]. Results are reported as percentage of DNA in the comet tail or tail intensity for each cell expressed as means of the median at different experimental points.

## Statistical analysis

Data are presented as box plots where mean, median and quartile values were calculated. The central line of the box and bars represents respectively the median, 50% and 25% of the measurements for each parameter. Differences between samples after intense exercise or supplementation were analyzed using paired Student’s t-test or one-way ANOVA analysis for multiple analysis taking into account the recovery phase. In this case, Post-hoc analysis of differences between samples was calculated using Tukey's honestly significant difference method. Values of *p* ≤ .05 are considered statistically significant and *p*-values ≤.01 are considered highly significant.

## Results

### Physical performance

**Figure 2.**
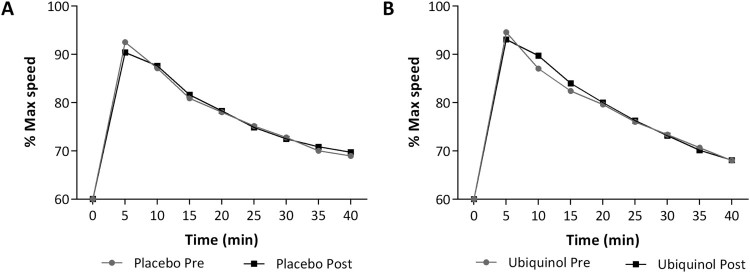
Timecourse of the speedrate of runners. Speed is expressed as percentage of maximal speed recorded before supplementation (gray) and after treatment (black). Placebo (A) or 200 mg of ubiquinol (B) for 1 month.

**Figure 3.**
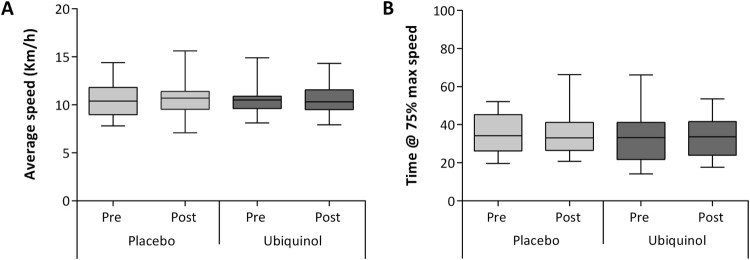
Distribution of average speed (A) and total time of run conducted at values equal and above 75% of the maximal speed (B). For both parameters distribution of data is represented for each of the four experimental phases.

Figure 2 presents the average speeds expressed as percentage of maximal speed at baseline (gray) and after 1 month of treatment (black). In particular, data of patients before and after placebo (Figure 2(A)) or 200 mg of ubiquinol (Figure 2(B)) are reported. A slight progressive decline in maximal speed was observed in each single session of intense exercise, with values approximately at 70% of the maximal initial speed after 40 min. After 5 and up to 15 min of running, subjects treated with ubiquinol seemed to keep a higher running pace (Figure 2(B)). No significant differences were observed in terms of ability of ubiquinol to allow a prolonged run at higher speed. Moreover other indicators of physical performance, calculated in terms of endurance indexes were not influenced by ubiquinol treatment. In particular distributions of average speed (Figure 3(A)) and time required to reach 75% of the max speed (Figure 3(B)) did not show any significant differences.

### Hematochemical and coenzyme q_10_ determination

#### Effect of a single bout of intense exercise before supplementation

**Figure 4.**
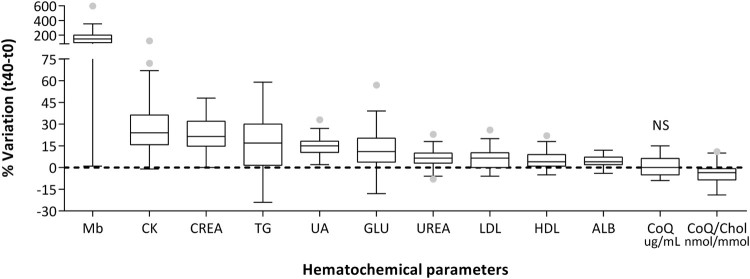
Hematochemical parameters expressed as percentage of variation after a single session of intense exercise. Notes: Mb: myoglobin; CK: creatine kinase; CREA: creatine; TG: triglycerides; UA: uric acid; GLU: glucose; UREA: urea; LDL: low density lipoproteins; HDL: high-density lipoproteins; ALB: albumin; CoQ plasma content (µg/mL) and lipoprotein content (nmol CoQ/mmol Cholesterol). Data are expressed as box plot of % variation pre-post session. All variations are highly significant (*p* < .01) except for CoQ plasma content, NS: not significant.

Figure 4 reports the percentage of variation in the levels of different hematochemical indexes produced by a single session of intense exercise. We can observe a significant concentration in all the parameters analyzed, except for Coenzyme Q_10_. In fact, plasma content expressed as µg/mL was unaltered while CoQ_10_ lipoprotein content in over 75% of the population studied decreased. Increases in concentrations of these analytes are mainly associated with a decrease in hematic volume following enhanced water loss by plasma, a process known as *ispessitio sanguis.* In some cases this effect is further enhanced by an active release in the hematic torrent, as in the case of creatine kinase (CK) and of myoglobin (Mb) that were chosen as indicators of muscular damage. In particular, the median percentage of variation of this last index increased up to two orders of magnitude, while for the other parameters considered the increase was between 4 and 20 fold.

#### Effect of single bout of intense exercise after supplementation

##### Creatine kinase and myoglobin plasma levels

**Figure 5.**
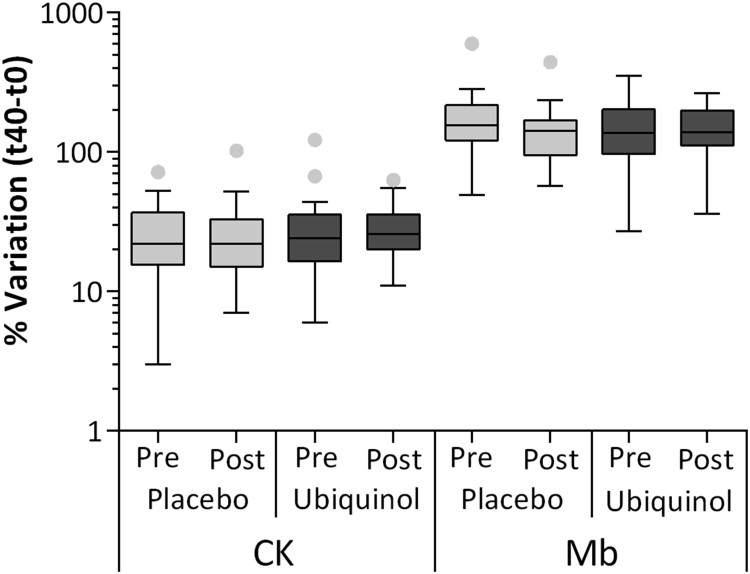
Creatine kinase (CK) and Myoglobin (Mb) level at the end of session of intense exercise (t40–t0) and after placebo or 200 mg of ubiquinol supplementation for 1 month. Note: Data are expressed as % of CK and Mb variation and they are represented as box plot diagram.

The hematochemical analytes whose plasma concentration varied the most after the running effort were myoglobin (Mb) and creatine kinase (CK), representing markers of muscular damage. In particular CK showed an increase comparable with that of other indexes (Figure 5) and on average, placebo pre= 26% ± 3%, post = 27% ± 6% and ubiquinol pre = 30% ± 7%, post = 29 ± 3%. The increase in Mb was more pronounced (Figure 5) and on average, placebo pre= 170% ± 34%, post= 149% ± 21% and ubiquinol pre= 154% ± 21%, post= 150% ± 14%. Ubiquinol treatment was not able to significantly influence the increase in both hematochemical indexes of muscular damage.

#### Total plasma coenzyme q_10_ content and its oxidative status

**Figure 6.**
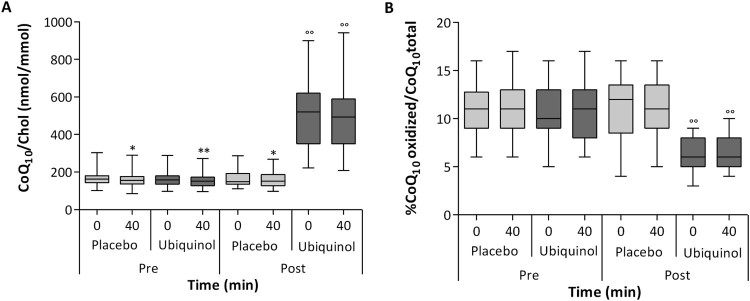
Plasma levels of coenzyme Q_10_ normalized to cholesterol levels (A) and percentage of oxidized coenzyme Q_10_ (B) before and after both session of intense exercise and placebo or 200 mg of ubiquinol supplementation for 1 month. Notes: Data are respectively expressed as CoQ10 nmol/cholesterol mmol and % of CoQ10 oxidized/CoQ10 total and they are represented as box plot diagram. *Significantly different from t0; °Significantly different from pre-supplementation values; *p < .05; **/°°*p* < .01.

In all experimental phases except for post ubiquinol supplementation, a statistically significant decrease was confirmed in lipoprotein CoQ_10_ content following 40 min of physical exercise at 85% maximal HR (Figure 6(A)). In particular, when comparing T0 and T40 values, plasma CoQ_10_ normalized by cholesterol content decreased significantly by 3.6–4.5% (*p* < .05). Ubiquinol supplementation (200 mg/day for 1 month) was able to remarkably enhance LDL CoQ_10_ content (+222%; *p* < .01). This increase efficiently counteracted CoQ_10_ deprivation induced by exercise. In fact, in ubiquinol treated subjects, a slight 1.9% non-significant decrease was observed following a single bout of physical exercise.

Differently from coenzyme Q_10_ plasma content, a single bout of intense exercise did not affect the oxidative status of CoQ_10_ in each of the four experimental sessions when comparing the percentage of oxidized CoQ_10_ at T0 and T40 (Figure 6(B)). On the other hand, ubiquinol supplementation produced a highly significant (*p* = .0001) improvement in plasma CoQ_10_ oxidative status in volunteers, presenting a percentage of oxidized CoQ_10_ on average of 6.5% both before and after the 40 min run, while baseline and placebo supplemented subjects had on average 11% of oxidized CoQ_10_ independently of physical exercise.

### Modulation of plasmatic and cellular oxidative status before and after supplementation

#### Paraoxonase and arylesterase activities of paraoxonase 1 (PON-1)

**Figure 7.**
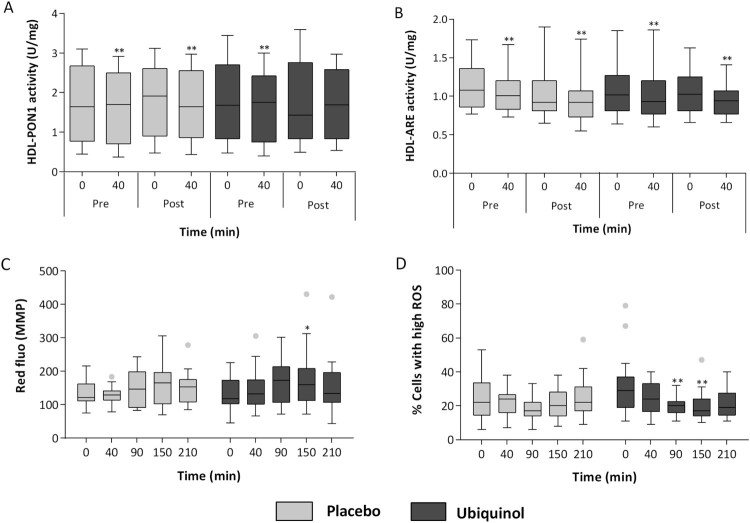
Paraoxonase (PON1) (A) and arylesterase (ARE) activity (B) normalized to high-density lipoprotein (HDL) before and after both sessions of intense exercise and placebo or 200 mg of ubiquinol supplementation for 1 month. Mean fluorescence intensity of MitoSense Red proportional to mitochondrial membrane potential (C) and percentage of cells with high intracellular ROS content (D) after both a single session of intense exercise and during recovery (t90–150–210 min) and after placebo or 200 mg of ubiquinol supplementation for 1 month. Notes: Data are respectively expressed as Unit/milligrams of high-density lipoprotein and red or green fluorescence of cation probe and they are represented as box plot diagram. *Significantly different from t0; *p < .05; **p < .01.

Antioxidant activities of PON1, in terms of PON1 paraoxonase activity (Figure 7(A)) and PON1 arylesterase (ARE) activity (Figure 7(B)), were evaluated and normalized by high-density lipoprotein content (Unit/milligram of high density of lipoprotein) to take into account their different concentrations in plasma following exercise. A single bout of intense exercise was able to decrease, in a highly significant manner both paraoxonase and arylesterase activities. In particular, at baseline and following placebo supplementation, both paraoxonase activity and arylesterase activity (_var0–40_ U/mg HDL) decreased on average by 7% (*p* < .01). In ubiquinol supplemented subjects, paraoxonase activity was not affected by physical exercise (var_0–40_=–4.5%; *p* = .45) (Figure 7(A)). However, ubiquinol did not counteract the highly significant arylesterase activity decrease in the same conditions (Figure 7(B)) (–10%; *p* < .01).

### Mitochondrial membrane potential

Mitochondrial membrane potential in PBMCs remained substantially unchanged (Figure 7(C)) both following placebo or ubiquinol treatment. Nonetheless between 90 and 210 min, during which time cells were allowed to recover *ex-vivo* at 37°C in complete medium, a slight hyperpolarization was observed. The extent of this variation was not significant after placebo, while in the same subjects taking ubiquinol a significant hyperpolarization was reached after 150 min (*p* < .05) and to a lesser extent at 90 min (*p* = .07).

### PBMCs cytosolic ROS and oxidative DNA damage

Intracellular ROS were monitored before and after the 40 min run at 85% of maximal HR and in the *ex-vivo* recovery phase up to 210 min. Immediately after the exercise stimuli no significant variations were observed both in placebo and ubiquinol treated subjects (Figure 7(D)). However, in the recovery phase, a distinct ROS scavenging effect was evident: a highly significant decrease in intracellular ROS was observed in the ubiquinol treated group at 90 and 150 min (–37.4% and –35.5%; *p* < .01). Moreover DNA oxidative damage was evaluated using the comet assay and no significant variation was observed either after a single bout of intense exercise or during the recovery phase up to 210 min both following placebo or ubiquinol treatment (data not shown).

## Discussion

The present study was aimed at evaluating the variation in plasma and cellular parameters associated with oxidative stress induced by a single bout of intense exercise either following placebo or 200 mg/day of ubiquinol for 1 month. At plasma level, the study highlighted the fact that while the main plasma components increased when compared before and after a 40 min run at 85% of the maxHR, coenzyme Q_10_ content normalized by total cholesterol significantly decreased but did not undergo any alteration in its oxidative status. CoQ_10_ is present in plasma lipoproteins, mainly as ubiquinol where it exerts an antioxidant function. Lipoproteins are also a means of transport of CoQ_10_ to tissues like of other lipid components [33]. Our results indicate that intense exercise produces a significant depletion in lipoprotein CoQ_10_ content, that likely corresponds to an enhanced request by tissue compartments. In support of this hypothesis, on average a lower plasma CoQ_10_ content in trained subjects compared to sedentary ones has been previously documented [34–37] and a positive correlation between muscular CoQ_10_ content and exercise capacity has been observed [38,39]. Moreover, it is also known that statin treatment, a widely used hypocholesterolemic drug targeting cholesterol and also CoQ_10_ synthesis, has been related to impaired exercise capacity [40,41] as well as to an increased incidence of intolerance in athletes and physically active patients [42,43] which further supports an increased CoQ_10_ requirement in physical activity. Nonetheless some authors report a milder effect of statins with no significant repercussion on physical performance [44] or on incidence of exercise induced injuries [45]. These discrepancies can be interpreted by considering the differences on individual muscular CoQ_10_ status influenced by age, gender, metabolic and genetic background [46,47]. Moreover, regular training may have a role in preconditioning the muscle, supporting mitochondrial biogenesis and aerobic metabolism, thus limiting statin induced side-effects.

Our data highlight how the rapid dynamics of this depletion lead to strengthening the concept that adequate CoQ_10_ status is particularly relevant in active subjects. Ubiquinol supplementation in our experimental model was shown to efficiently curb exercise-induced CoQ_10_ depletion, proving its use as an efficient method to optimize CoQ_10_ status in physically active people. Ubiquinol is the reduced and active form of Coenzyme Q_10_ and, although our organism is able to efficiently reduce endogenous as well as dietary ubiquinone, we chose to test ubiquinol as a supplement in light of its improved bioavailability [28,48,49] and in the proper status to exert its antioxidant function without requiring any reductive steps. Supplementation produced a highly significant 4-fold increase in lipoprotein CoQ_10_ content that lowered by 50% the extent of exercise-induced depletion. Post exercise CoQ_10_ plasma content as a result remained well above the physiological un-supplemented levels. Surprisingly, no acute variation in CoQ_10_ oxidative status was observed after a single bout of physical exercise, a parameter that might seem more easily affected compared to the total content. However, this data could be interpreted in light of the fact that oxidative stress induced by a single bout of intense exercise is minor in trained young and healthy individuals where antioxidant and reductive mechanisms are effective and able to counteract oxidative insults.

Under our experimental settings, despite the fact that coenzyme Q_10_ oxidative status was unaltered, intense exercise was associated with a decrease in PON-1 enzymatic activities both in the paroxonase and arylesterase ones at baseline and in placebo supplemented patients. In fact, PON-1 is associated with HDL and it exerts an important anti-inflammatory and antioxidant role [50]. In particular PON1 is able to protect lipoproteins (HDL and LDL) and cell membranes against lipid peroxidation and recently several studies have outlined its role as an antiatherogenic agent counteracting LDL oxidation [51,52]. PON-1 modulation by physical exercise is a debated argument and plentiful evidence [53–56] indicates that regular physical training is able to upregulate HDL associated PON-1 activity contributing to the adaptive antioxidant response with the likely involvement of specific transcription inducers [57]. On the other hand strenuous exercise could promote radical formation and lipid peroxidation, known to be associated with a decrease in PON1 activity following oxidation of PON1 free sulfhydryl groups [58]. Moreover, an intense bout of physical exercise might promote a transfer of paraoxonase from HDL to cellular plasma membranes. In fact, Deakin et al. [59] showed that PON1 can be transferred from lipoproteins into the cell membrane and retain its activity, extending its protective functions outside the lipoprotein environment. Both mechanisms are likely to be associated in our experimental model. On this issue, animal [60,61] and human data [62,63] have provided conflicting results on the extent of exercise-mediated detrimental effects on HDL-associated PON-1 activity, since modulation might be influenced by the intensity of exercise stimuli but also by the training status of individuals as well as by their genetic background, specifically in relation to PON1 polymorphisms [64,65]. This complex scenario could explain the high variability observed in HDL-normalized PON1 paraoxonase activities reported in Figure 7(A). On the other hand the evaluation of arylesterase activity (Figure 7(B)), considered a measure of PON1 enzyme quantity, shows a lower variability [66]. As a result the data distribution within each phase is far less scattered for arylesterase.

Our data show that ubiquinol supplementation was able to prevent the exercise-induced paraoxonase activity decrease, but not the arylesterase one. The effect on paraoxonase activity, being strongly modulated by oxidative stress, might indicate an improved oxidative status of HDL and consequently a minor decrease in its activity that was spared by the antioxidant effect of ubiquinol present in greater amounts in lipoproteins of the supplemented subjects. This data highlights the synergistic activity of ubiquinol with other antioxidant systems in lipoproteins. In this respect, Tsakiris et al. [67], have shown that in basketball players arylesterase activity significantly decreased after a single bout of physical exercise, in agreement with our data. In that study however, one month supplementation with α-tocopherol was able to prevent the decrease in arylesterase activity. Both data suggest a beneficial effect of lipid antioxidant supplementation in limiting exercise-induced oxidative damage to PON-1. The differences observed might be related to the type of antioxidant used but also to the biochemistry of the subjects involved and the type of exercise model, which in this case was routine training rather than a single bout of intense exercise.

In an attempt to evaluate at the cellular level the effect of exercise, alone or in association with intense exercise, we isolated PBMCs to measure mitochondrial membrane potential and intracellular ROS levels. Although PBMCs may not be representative of the effects generated by intense exercise on skeletal muscle, in our experimental design they constituted a compromise between invasive procedure and evaluation of mitochondria containing cells that could provide some indications on the effect of ubiquinol at a cellular level. In fact, some authors have shown that physical activity and exercise have profound effects on the immune system [68,69].

Mitochondrial membrane potential in PBMCs in our experimental model did not seem to be affected by 40 min intense exercise, however a moderate increase in mitochondrial membrane potential was observed during the recovery phase *ex-vivo* between 90 and 150 min. Nonetheless, the extent of this increase was significant only in PBMCs from ubiquinol supplemented subjects after 150 min, which could be explained by the bioenergetic effect of this molecule. This increase in PBMCs may indicate an enhanced metabolic demand of cells following exercise that could in part reflect the behavior of more physiologically relevant tissues. In this respect, it has been shown that following exercise, intramuscular triglyceride (IMTGs) stores are reduced by 60%, in particular in type I muscle fibers suggesting an enhancement of mitochondria related catabolic processes [70,71].

Also for intracellular ROS, no significant differences were observed immediately after a single bout of intense exercise, although a slight decrease in the distribution is detectable suggesting possible cellular antioxidant adaptive responses. During the recovery phase, improvement in the antioxidant status is also evident at 90 and 150 min, significant only in ubiquinol supplemented subjects, further supporting a positive modulation of antioxidant defenses triggered by exercise stimuli. This may not be due to ubiquinol enrichment *per se* exerting a direct radical scavenging activity, but to improved signaling underlying antioxidant responses upregulated by ubiquinol. This is a critical issue because some antioxidants may decrease the ergogenic activity of exercise training by quenching oxidative stimuli at the basis of adaptive response [19,20,72]. However, ubiquinol is a particular molecule endowed with both antioxidant properties but also critical for mitochondrial bioenergetics. Recent reports have highlighted that differently from other antioxidants, ubiquinol is able to promote Nrf2 translocation and hence to increase positive stimuli following physical exercise, while inhibiting the NFkB inflammatory pathway [65,73–75].

Limitations of the study include high inter-individual variability and relatively low levels of variation in myoglobin and creatine kinase induced by the exercise protocol chosen. Therefore we cannot exclude that the positive effect of ubiquinol in this respect could have been amplified in the presence of a higher threshold of stress, as demonstrated by Sarmiento et al. [76]. In fact, in the present study we used a high intensity, mainly aerobic and endurance exercise, while the study by Sarmiento et al. implemented an aerobic and anaerobic resistance exercise at high intensity (60–70% dynamic maximum force). As a result, the markers of muscular damage were substantially more elevated post-exercise in their study.

## Conclusions

The present study has shown a rapid and significant decrease in plasma ubiquinol levels, in particular in terms of lipoprotein CoQ depletion, following a single bout of intense exercise. Moreover, ubiquinol was found to act in cooperation with endogenous systems enhancing plasmatic and cellular antioxidant defenses in PBMC. Despite this, ubiquinol supplementation in our experimental model was not able to improve indexes of physical performance or prevent enhancement of markers of muscular damage induced by exercise, contrary to other evidence in the literature. Moreover, besides the described antioxidant role, ubiquinol supplementation provided a buffering effect on plasma CoQ content highlighting the relevance of oral supplementation in trained athletes to balance exercise-associated depletion.
